# The effect of vitamin D on disease activity, fatigue and interferon signature gene expression in systemic lupus erythematosus

**DOI:** 10.31138/mjr.28.3.127

**Published:** 2017-09-29

**Authors:** Rosalie Magro, Andrew A. Borg

**Affiliations:** Rheumatology Department, Mater Dei Hospital, Msida, Malta

**Keywords:** Lupus Erythematosus, systemic, vitamin D, fatigue, gene expression

## Abstract

Systemic Lupus Erythematosus (SLE) is multi-system autoimmune disorder, whose pathogenesis involves several cascades that lead to the production of interferon alpha, which then mediates the manifestations of the disease. In SLE, the overexpression of interferon regulated genes, produce a unique interferon signature. This has a positive correlation with disease activity. Vitamin D deficiency is highly prevalent in SLE; the role of vitamin D in the course and prognosis of SLE is unknown. Vitamin D deficiency has been associated with a higher disease activity in SLE. Fatigue is also highly prevalent in SLE; its aetiology is multi-factorial. There is limited evidence on the relationship between vitamin D, fatigue and interferon signature gene expression. Further studies on this will establish whether treatment of vitamin D deficiency in SLE, has any significant effect on the level of fatigue and disease activity, and whether this could be due to the suppression of interferon signature gene expression.

## INTRODUCTION

Systemic Lupus Erythematosus (SLE) is a multi-system autoimmune disorder with a reported prevalence of 20 to 50 per 100,000 people in Europe. It affects females up to nine times more frequently than males. Sixty-five percent of patients with SLE have a disease onset between the ages of 16 and 55. The aetiology includes genetic, epigenetic and environmental factors (including UV light, infections, drugs), that lead to an irreversible break in immunological tolerance. SLE is associated with the presence of autoantibodies, predominantly those that target double-stranded DNA (dsDNA) and extractable nuclear antigens. The variable clinical features involve multiple systems including the skin, musculoskeletal, renal, neurological, haematologic, cardiovascular and respiratory systems.^1^

The underlying pathogenesis in SLE is initiated and propagated by the impaired clearance of apoptotic cells and the loss of tolerance to self-antigens. Plasmacytoid dendritic cells produce type 1 interferon (IFN) cytokines that have a leading role in the development of SLE.^2–4^ Type 1 IFNs promote the production of various stimulators by myeloid dendritic cells. These include the production of B lymphocyte stimulator and a proliferation-inducing ligand, which are involved in the survival of autoreactive B cells, leading to the generation of pathogenic autoantibodies. Moreover, type 1 IFNs induce the production of interleukin(IL)-6 and IL-23 that promote T helper 17 cell responses. This leads to the production of IL-17 that promotes B cell hyperreactivity, as well as tissue inflammation and damage by recruiting neutrophils, macrophages and lymphocytes.

Vitamin D is a fat-soluble vitamin, which is present in few foods. The main source of vitamin D is its synthesis in the skin upon absorption of ultraviolet B radiation by 7-dehydrocholesterol. It then undergoes hydroxylation in the liver to 25-hydroxyvitamin D; and then further hydroxylation to 1,25-hydroxyvitamin D, which is the active form. Vitamin D promotes calcium absorption in the gastrointestinal tract and maintains adequate serum calcium and phosphate concentrations. It is also important for bone growth and bone remodeling.^5^ Vitamin D receptor is present in most cells, and vitamin D can regulate the transcription of over 200 genes. It thus has multiple roles, including modulation of cell growth, neuromuscular and immune function, and reduction of inflammation.^6^

Vitamin D deficiency is defined as serum 25-hydroxyvitamin D concentrations below 20ng/mL. Its prevalence in adults in Europe ranges from 34% to 67%.^7^ Vitamin D deficiency is more prevalent among SLE patients, possibly due to sun avoidance and renal impairment in lupus nephritis.^8^ The expression of vitamin D receptors by a variety of cells belonging to the innate and adaptive immune systems (including macrophages, dendritic cells, T cells and B cells) has created interest with regards to the role of vitamin D in the pathogenesis of SLE.^9^ It is still unknown whether vitamin D deficiency alters the course and prognosis of SLE.

## AIM AND METHODOLOGY

The aim of this review is to summarise the available evidence on the relationship between vitamin D, fatigue, disease activity and interferon signature gene expression in SLE. MEDLINE/PubMed was searched for articles published in English up to July 2016, using the MeSH terms “vitamin D”, “fatigue”, “interferon”, “gene expression” and “systemic lupus erythematosus”. Additional papers were selected from the references included in these articles. The abstracts were reviewed, and when considered relevant, the full text was reviewed for inclusion in this paper.

### Vitamin D and Interferon Signature Gene Expression

Studies on gene expression profiling have shown that in the peripheral blood of SLE patients, there is overexpression of type 1 interferon-regulated genes (IFN signature). This has a positive correlation with disease activity.^10^ A meta-analysis has identified 12 such genes that are either interferon induced or interferon regulated.^11^ The overexpression of the IFN signature distinguishes SLE from other autoimmune conditions such as antiphospholipid syndrome, rheumatoid arthritis and multiple sclerosis. The expression of the IFN signature could be used for diagnosis and monitoring of SLE. Moreover, it provides targets that could be used for the development of new therapies for SLE.

A cross-sectional case-control study including 32 SLE patients showed that SLE patients with vitamin D deficiency had a significantly higher mean serum IFN-α activity than those without vitamin D deficiency.^12^ This was confirmed in another case-control study that showed that in SLE patients, serum 25-hydroxyvitamin D significantly correlated in an inverse manner with plasma IFN-α and levels of IFN-α gene expression.^13^ In a randomized controlled trial, supplementation of vitamin D 2000IU or 4000IU daily to SLE patients with vitamin D deficiency, failed to demonstrate an effect on IFN signature gene expression at 12 weeks.^14^ However, a loading dose of vitamin D was not used in this trial, and 48.5% of patients receiving vitamin D did not achieve levels of 25-hydroxyvitamin D above 30ng/ml. The authors concluded that higher levels of 25-hydroxyvitamin D, sustained for a longer duration, may be required to suppress IFN signature gene expression (**Table 1**).

**Table 1.** Table showing details of studies that have assessed the relationship between vitamin D level and interferon signature gene expression in SLE.

**Study (year)**	**Country**	**Study Design**	**Study Population**	**Association between vitamin D and IFN signature gene expression**
**Ritterhouse LL et al. ^12^ (2011)**	United States	Cross-sectional case control	32 SLE 32 controls	*p*=0.02^*^
**Mandal M et al.^13^ (2014)**	India	Cross-sectional case control	129 SLE 100 control	*p*=0.0009^*^ r=−0.45
**Aranow C et al.^14^ (2015)**	United States	Randomised, placebo controlled trial	57 SLE	*p*=0.77

* statistically significant results

### Vitamin D and Disease Activity

A number of studies have looked into the relationship between vitamin D and disease activity with conflicting results. Most studies are cross-sectional studies; some are prospective studies, and few are randomized controlled trials. All studies used a validated scoring system to measure disease activity, including Systemic Lupus Erythematosus Disease Activity Index (SLEDAI), Systemic Lupus Erythematosus Activity Measure (SLAM), European Consensus Lupus Activity Measurement (ECLAM) and British Isles Lupus Activity Group (BILAG). Twenty studies reported a significant inverse association between vitamin D level and lupus disease activity.^13,15–33^ Nineteen studies failed to show such a significant relationship.^14,34–51^

A systematic review that looked into the clinical significance of vitamin D in SLE concluded that there is a significant inverse association between vitamin D and disease activity measurements (including SLEDAI, high anti-dsDNA titres and low complement levels).^9^ The level of vitamin D was not associated with organ damage. Vitamin D deficiency has also been associated with renal,^8,45^ and cardiovascular disease in SLE.^27,52^ A meta-analysis on the correlation between serum level of vitamin D and lupus disease activity confirmed the inverse association between these two factors.^50^ It included 11 articles that presented this association with Pearson correlation tests.^17,19–21,25,28,30,48,50^

The pooled Pearson correlation between disease activity and vitamin D was −0.365 (95% confidence interval: −0.536, −0.165). However, other studies that did not report the Pearson correlation coefficient were not included; these included studies that did not find a correlation between vitamin D and disease activity.

### Vitamin D and Fatigue

There is not a consensus definition of fatigue in the literature but fatigue is often described as the overwhelming sensation of weakness, lack of energy, whole-body tiredness or exhaustion that is chronic, typically unrelated to over-exertion and poorly relieved by rest. Fatigue is the most prevalent symptom in SLE, as it is present in up to 90% of patients;^53^ it is considered to be the most disabling symptom in around half of the patients.^54^ The aetiology of fatigue is multi-factorial and includes co-morbid conditions (such as depression,^55–57^ sleep disorder and fibromyalgia^58^), and behavioural factors such as physical activity.^59–60^ Conflicting data is present with regards to the association of disease activity and fatigue; in some studies a strong association was found^55,57,61,62^ and in others a weak or no association was present.^53,56,63^ In 2007, the Ad Hoc Committee on Systemic Lupus Erythematosus Response Criteria for Fatigue reviewed fifteen different instruments that have been used to measure fatigue in SLE.^64^ The instrument recommended was the Fatigue Severity Scale (FSS), a 9-item scale with a 1–7 possible score in each item.

There is conflicting evidence on the relationship between vitamin D level and fatigue in adults with SLE (**Table 2**). Four studies have been identified that have specifically looked into this; three cross-sectional studies and one prospective study.^36,37,43,65^ Out of these four studies, only the prospective study by Ruiz-Irastorza et al*.* showed a significant inverse association between vitamin D level and fatigue. In these studies, the recommended instrument to measure fatigue, the FSS, was used only in the study by Stockton et al. In the other three studies, the visual analogue scale (VAS) was used.

**Table 2.** Table showing details of studies that have assessed the relationship between vitamin D level and fatigue in SLE.

**Study (year)**	**Country**	**Study Design**	**Study Population**	**Fatigue measure**	**Association between vitamin D and fatigue**
**Ruiz-Irastorza G et al.^36^ (2008)**	Spain	Cross-sectional cohort	92 SLE	VAS	*p*=0.08
**Ruiz-Irastorza G et al.^37^ (2010)**	Spain	Prospective cohort	80 SLE	VAS	*p*= 0.015^*^
**Fragoso TS et al.^43^ (2012)**	Brazil	Cross-sectional case control	78 SLE 64 controls	VAS	*p*=0.808
**Stockton KA et al.^65^ (2012)**	Australia	Cross-sectional case control	24 SLE 21 controls	FSS	*r*= −0.12
**Lima L et al. ^33^ (2016)**	Brazil	Randomised, placebo controlled trial	40 juvenile SLE	K-FSS	*p*= 0.008^*^

* statistically significant results

A randomized double-blind placebo controlled trial analysed the effect of vitamin D supplementation in juvenile-onset SLE.^33^ 45 patients up to 25 years old, who had symptoms before 16 years of age, were included. In this 24 week trial, the patients were randomised to receive oral cholecalciferol 50,000IU/week or placebo. A per-protocol analysis showed a significant reduction of fatigue interfering in social life (a component of the Kids Fatigue Severity Scale) in the treatment group, compared to the control group. Similarly, a significant improvement in disease activity (measured by SLEDAI, ECLAM and anti-dsDNA) was noted in the treatment group.

## CONCLUSION

The various studies included in this review are heterogeneous in terms of their study design and the characteristics of the included patients. Evidence from multiple studies, including a systematic review and meta-analysis, has shown that vitamin D deficiency in SLE is associated with a higher disease activity. This could be due to the immunological effect exerted by vitamin D, via the vitamin D receptor, to suppress auto-immunity. Moreover, vitamin D deficiency could be the result of active disease, particularly in lupus nephritis. In this case, renal impairment results in decreased hydroxylation of vitamin D leading to decreased production of 1,25-hydroxyvitamin D, which is the active form. Fatigue in SLE is multi-factorial and it is associated with co-morbid conditions (e.g. sleep disorder, depression, fibromyalgia) and behavioural factors such as decreased physical activity. There is conflicting evidence with regards to the relationships between fatigue and disease activity, and fatigue and vitamin D level. No studies looking into the relationship between IFN signature gene expression and fatigue could be identified.

Further well-designed studies looking into these factors in SLE are required; particularly using the recommended fatigue severity scale to measure fatigue, as well as ensuring adequate vitamin D supplementation in prospective therapeutic studies. In particular the effect of treating vitamin D deficiency, on the level of fatigue and disease activity, need to be addressed. If these are found to improve, then screening for vitamin D deficiency should be recommended in all SLE patients. Moreover, the relationship between disease activity and fatigue needs to be studied. This could provide a guide as to whether tighter control of disease activity in clinical practice, may be reflected in an improved level of fatigue.

The unique overexpression of the IFN signature in SLE is related to the underlying disease activity. Further studies are required to elucidate whether treatment of vitamin D deficiency in SLE suppresses the IFN signature gene expression, possibly by the role of the vitamin D receptor present in plasmacytoid dendritic cells. This could provide an explanation with regards to the underlying mechanism by which vitamin D supplementation reduces disease activity in SLE. Another issue that needs to be addressed in future research, is the relationship between the IFN signature gene expression and fatigue; and whether the IFN pathway may provide novel targets in the management of fatigue in SLE.

## ABBREVIATIONS

BILAG: British Isles Lupus Activity Group
dsDNA: double-stranded DNA
ECLAM: European Consensus Lupus Activity Measurement
FSS: Fatigue Severity Scale
IFN: interferon
IL: interleukin
MeSH: medical subject headings
SLAM: Systemic Lupus Erythematosus Activity Measure
SLE: Systemic Lupus Erythematosus
SLEDAI: Systemic Lupus Erythematosus Disease Activity Index
VAS: visual analogue scale
